# Effect of Aerobic Exercise on Blood Glucose Among Those with Prediabetes: A Systematic Review and Meta-Analysis

**DOI:** 10.3390/life15010032

**Published:** 2024-12-30

**Authors:** Tianyi Jiang, Zichen Ye, Qu Lu, Peixia Cheng, Qi Gao

**Affiliations:** 1 Department of Epidemiology and Health Statistics, School of Public Health, Capital Medical University, Beijing 100069, China; jiangtianyi@i.pkuschool.edu.cn; 2 School of Population Medicine and Public Health, Chinese Academy of Medical Sciences and Peking Union Medical College, Beijing 100730, China; ye18700579760@163.com; 3 School of Health Policy and Management, Chinese Academy of Medical Sciences and Peking Union Medical College, Beijing 100730, China; qulu167@163.com; 4 Department of Maternal, Child and Adolescent Health, School of Public Health, Capital Medical University, Beijing 100069, China

**Keywords:** prediabetes, aerobic exercise, RCT, meta-analysis, systematic review

## Abstract

Prediabetes, a state between normoglycemia and diabetes, is increasingly affecting population health; thus, it should not be overlooked. This systematic review and meta-analysis aimed to explore the efficacy of aerobic exercise on blood glucose indicators among those with prediabetes. Five databases, including PubMed, Embase, the Cochrane Library, Web of Science, and CINAHL, were searched up to September 2024 to identify randomized controlled trials measuring the effect of aerobic exercise on blood glucose levels among individuals with prediabetes. Data on fasting blood glucose (FBG), 2-h postprandial plasma glucose (2hPG), and hemoglobin A1c (HbA1c) were extracted. Subgroup analyses were conducted based on intervention duration, weekly exercise duration, and volume. In total, 2518 relevant records were initially retrieved, and 16 studies were included in this systematic review, with 14 providing sufficient data for meta-analysis. Compared to the control group, the weighted mean difference (MD) for FBG, 2hPG, and HbA1c was −1.33 (95%CI: −1.87, −0.89), −1.00 (95%CI: −1.60, −0.39), and −1.05 (95%CI: −1.49, −0.61), respectively. Subgroup analysis showed that pooled effect size for FBG, 2hPG, and HbA1c was greater in the subgroup subjected to ≥48 weeks of intervention compared to the subgroup subjected to ≤24 weeks of intervention, although only the difference in FBG was significant (*p* < 0.05). Weekly exercise longer than 180 min/week led to greater reductions in FBG, 2hPG, and HbA1c compared to weekly exercise shorter than 150 min/week, and only differences in 2hPG were not significant (*p* > 0.05). Total weekly exercise of 1314–1323 MET·min/week led to greater reductions in FBG and HbA1c levels compared to 975–1080 MET·min/week (*p* > 0.05). Aerobic exercise effectively decreases FBG, 2hPG, and HbA1c and controls blood glucose levels. The volume and duration of aerobic exercise are important factors affecting the reduction in blood glucose levels, exhibiting a positive correlation within a specific range. Aerobic exercise can serve as a viable therapeutic approach for reducing the risk of diabetes among individuals with prediabetes.

## 1. Background

Diabetes is a severe chronic metabolic disorder caused primarily by the lack of insulin secretion or impaired cellular response to insulin, which may lead to persistent hyperglycemia and undermine the function of several physiological systems, including the cardiovascular, nervous, and renal systems [1]. Diabetes has emerged as one of the top five most serious chronic diseases worldwide [2]. More than 537 million people aged 20–79 years suffered from diabetes as of 2021, with a prevalence rate of as high as 9.8%. The situation is expected to worsen, with the International Diabetes Federation forecasting 784 million cases and a prevalence rate of 11.2% by 2045 [3]. The escalating prevalence underscores the urgent need for the prevention and control of diabetes, emphasizing the importance of effective interventions to mitigate the booming challenges posed by this pervasive health problem.

Prediabetes, a state between normoglycemia and type 2 diabetes, encompasses impaired fasting glucose (IFG) and impaired glucose tolerance (IGT). It is a growing global health concern, characterized by elevated blood glucose levels that do not meet the threshold for diagnosing diabetes [4,5]. Prediabetes encompasses conditions such as IFG, which reflects an elevated fasting blood glucose level, usually between 5.6 to 6.9 mmol/L, and IGT, which is characterized by increased postprandial blood glucose levels two hours after a glucose challenge, usually ranging between 7.8 to 11.0 mmol/L, based on World Health Organization (WHO) criteria [6]. IFG and IGT are considered two types of prediabetes because they indicate early-stage dysfunction of glucose metabolism and significantly increase the risk of type 2 diabetes among individuals [7]. Although prediabetes affects adults of all ages, its prevalence increases with age. For example, data from 2017–2020 in the United States showed that 27.8% of adults aged 18–44 had prediabetes, whereas this proportion increased to 44.8% among those aged 45–64 and to 48.8% among adults aged 65 and older [8].

The progression of prediabetes to type 2 diabetes emphasizes the urgent need for effective interventions. Importantly, these conditions can be effectively managed, lowering blood glucose levels and reducing the risk of progression to diabetes [9]. Previous studies have shown that effective interventions for prediabetes include dietary adjustments, physical activity, and pharmacological treatment [10,11,12]. Among them, physical activity is considered to play a key role in preventing the progression of prediabetes to diabetes. Numerous health organizations have incorporated aerobic exercise into their public health recommendations and clinical guidelines. For instance, WHO recommends at least 150 min of moderate-intensity aerobic activity or 75 min of vigorous-intensity activity per week for adults, with additional benefits from increased duration and intensity [13]. Similarly, the American Diabetes Association encourages regular physical activity, including aerobic exercise, as part of a comprehensive approach to managing prediabetes and diabetes [14]. These guidelines are supported by a growing body of evidence demonstrating the efficacy of aerobic exercise in improving glycemic control. Moderate exercise enhances insulin sensitivity, lowers body weight, decreases blood glucose levels, and concurrently improves cardiovascular health. Additionally, aerobic exercise is highly recommended for managing prediabetes due to its low intensity, long duration, cost-effectiveness, broad applicability, and huge health benefits.

Despite the widespread implementation of aerobic exercise interventions among individuals with prediabetes, few studies summarized the effects of aerobic exercise on glycemic control among patients with prediabetes. To address this knowledge gap, we conducted a systematic review and meta-analysis of published randomized controlled trials (RCTs) to determine the efficacy of aerobic exercise in preventing prediabetes. This study not only provides more specific guidance for healthcare professionals but also offers concrete exercise recommendations for patients with prediabetes.

## 2. Methods

### 2.1. Protocol Registration and Study Design

The study protocol has been registered in the PROSPERO International Registry of Systematic Reviews (Registration Number CRD42024490348). This study followed the Preferred Reporting Items for Systematic Reviews and Meta-Analyses 2020 guidelines (PRISMA 2020) [15], as outlined in Table S1. No ethics approval or informed consent was needed for secondary analysis data.

### 2.2. Search Strategy

PubMed, Embase, the Cochrane Library, Web of Science Core Collection, and CINAHL were systematically searched from inception until 8 September 2024. The comprehensive search strategy for each database was developed jointly by two researchers and reviewed and approved by other experienced researchers. The detailed search strategies are available in Supplementary Material S2.

### 2.3. Eligibility Criteria

Inclusion criteria were as follows: (1) RCT with a two-arm design; (2) studies whose participants were diagnosed with prediabetes; (3) monitored exercise with specific details on frequency, intensity, and volume, meeting aerobic exercise standards, i.e., achieving 60–70% of the maximum heart rate; (4) outcome measures included FBG, 2-h postprandial plasma glucose (2hPG), or glycated hemoglobin A1c (HbA1c), etc. No language, regional, or publication restrictions were applied. After eliminating duplicate results, two researchers initially searched and screened the titles and abstracts of the retrieved records using EndNote 21 software based on predetermined criteria. Full-text reports were reviewed to determine whether they met the inclusion criteria. Disagreements were solved through discussion or consultation with a third researcher. 

### 2.4. Data Extraction

Two researchers independently extracted study characteristics and outcome measures using a pre-designed data extraction form. A third researcher resolved discrepancies. The following data were extracted from the included studies: (1) characteristics of studies (author name, publication date, or sample size); (2) sample characteristics (average age and gender percentages); (3) intervention and comparator characteristics (type of exercise, frequency, intensity, and intervention duration), and (4) outcome measures (HbA1c, FBG, or 2hPG). If the study included additional intervention groups (e.g., diet and resistance training) besides the aerobic and control groups, data were only extracted for the aerobic and control groups.

### 2.5. Quality Assessment

The revised Cochrane Risk of Bias Version 2 tool was employed to assess the quality of the included studies across five domains: randomization process, deviations from the intended interventions, missing outcome data, measurement of the outcome, and selection of the reported results [16]. The bias risk for each domain was determined as high, low, or having some concerns. The overall bias was deemed low if all five domains were classified as low risk of bias. If all five domains were not assessed as having a high risk of bias but there was a potential risk of bias in one of the domains, the overall assessment came with some concerns. If any domains were assessed as having a high risk of bias or multiple domains were categorized as having some concerns and the effect on the credibility of the study results was substantial, the overall assessment was regarded as having a high risk of bias. Two authors conducted the assessment, and discrepancies were resolved through discussion with a third researcher.

### 2.6. Statistical Analysis

Glucose levels are often reported as either milligrams per deciliter (mg/dL) or millimoles per liter (mmol/L). In this study, glucose values reported in mmol/dL were converted to mmol/L by multiplying them by 0.0555, as described in a previous study [17]. Standard deviation (SD) was estimated using Wan’s method 18 for outcome measures with quartile values. When outcome measures were reported as mean and 95% confidence interval (CI), they were converted to SD following the guidelines outlined in Chapter 7.7.3.2 of Cochrane’s Handbook [18]. Studies not reporting quartile SD, standard error (SE), or 95%CI were excluded from this meta-analysis. All results were harmonized into the format “mean ± standard deviation (M ± SD)” for data synthesis.

I^2^ statistic was used to evaluate heterogeneity among studies, with high heterogeneity defined as I^2^ greater than 50%. Subsequently, subgroup analyses and sensitivity analyses were conducted to explore potential sources of heterogeneity. Random-effect models were applied to address the high heterogeneity across studies. Publication bias was examined using funnel plots and Begg’s test.

Subgroup analyses were conducted for FBG, 2hPG, and HbA1c values based on intervention duration (12-week, 24-week, and ≥48-week), weekly exercise duration (100–150 min/week, 180 min/week, and ≥200 min/week), and weekly exercise volume (975–1080 MET·min/week and 1314–1323 MET·min/week). Based on the literature [19] and Calorie Expedience for Variety Exercises [20], the metabolic equivalent (MET) corresponding to the exercise mode was obtained and the weekly exercise volume was calculated. Meta-analysis was conducted only when two or more original studies were available in a subgroup. All data were analyzed using Stata (version 18.0). Differences with *p*-values less than 0.05 were deemed statistically significant, and all results were based on two-tailed tests.

## 3. Results

### 3.1. Study Selection and Characteristics of Eligible Studies

Initially, 2518 relevant records were identified by comprehensively searching five databases, of which 927 duplicates were removed using reference manager software. Then, 1591 studies were removed based on titles and abstracts. The full texts of 32 potentially eligible articles were reviewed to determine whether they met our inclusion criteria, and 16 studies were finally excluded due to the following reasons: 15 underwent interventions combining aerobic exercise with other measures, and 1 provided an unclear description of aerobic exercise. Finally, 16 articles were included in this systematic review, although only 14 provided sufficient data for meta-analysis (Figure S1).

This study included 1104 patients with prediabetes, with females accounted for 64.6% of participants (*n* = 713). Participants were mostly middle-aged and elderly, aged 40 to 65. Only one group of participants were adolescents aged 10 to 16 years. Physical exercise in the intervention groups mainly included aerobic dance, jogging, yoga, and brisk walking. Most studies set the frequency of exercise at three times per week with a single exercise duration of 60 min. Exercise intensity was predominantly described based on HRmax, with seven studies having training intensity between 60% and 70%. The intervention duration ranged from a minimum of 3 months to a maximum of 24 months (Table 1).

### 3.2. Effects of Aerobic Exercise on FBG

**Figure 1:**
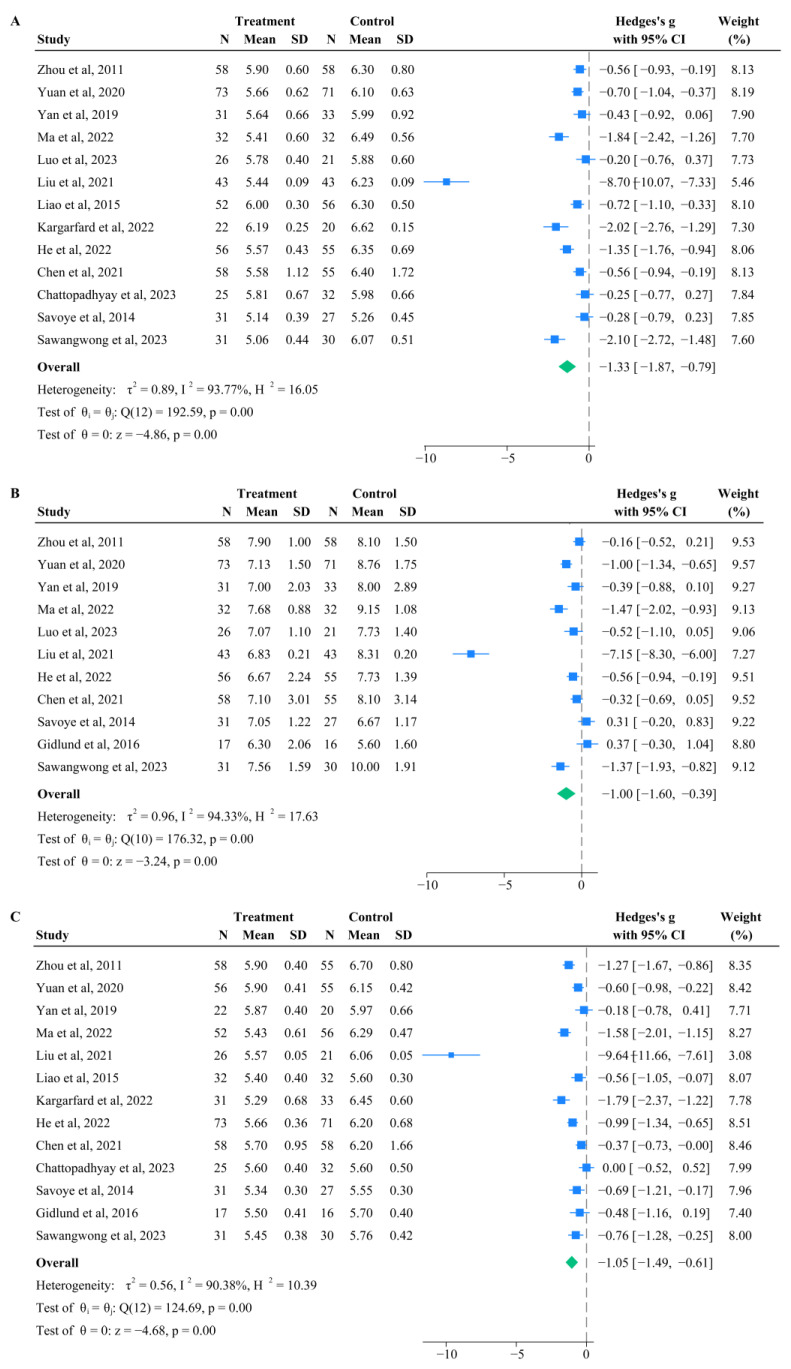
Summary estimate of pooled performance using forest plot [21,22,23,25,26,27,28,29,30,31,33,34,35,36]. (**A**) Forest plot of FBG; (**B**) Forest plot of 2hpg; (**C**) Forest plot of Hba1c. Note: The ‘blue squares’ represent the effect size of each individual study and the ‘green diamond’ represents the overall pooled result for the entire group.

**Figure 2:**
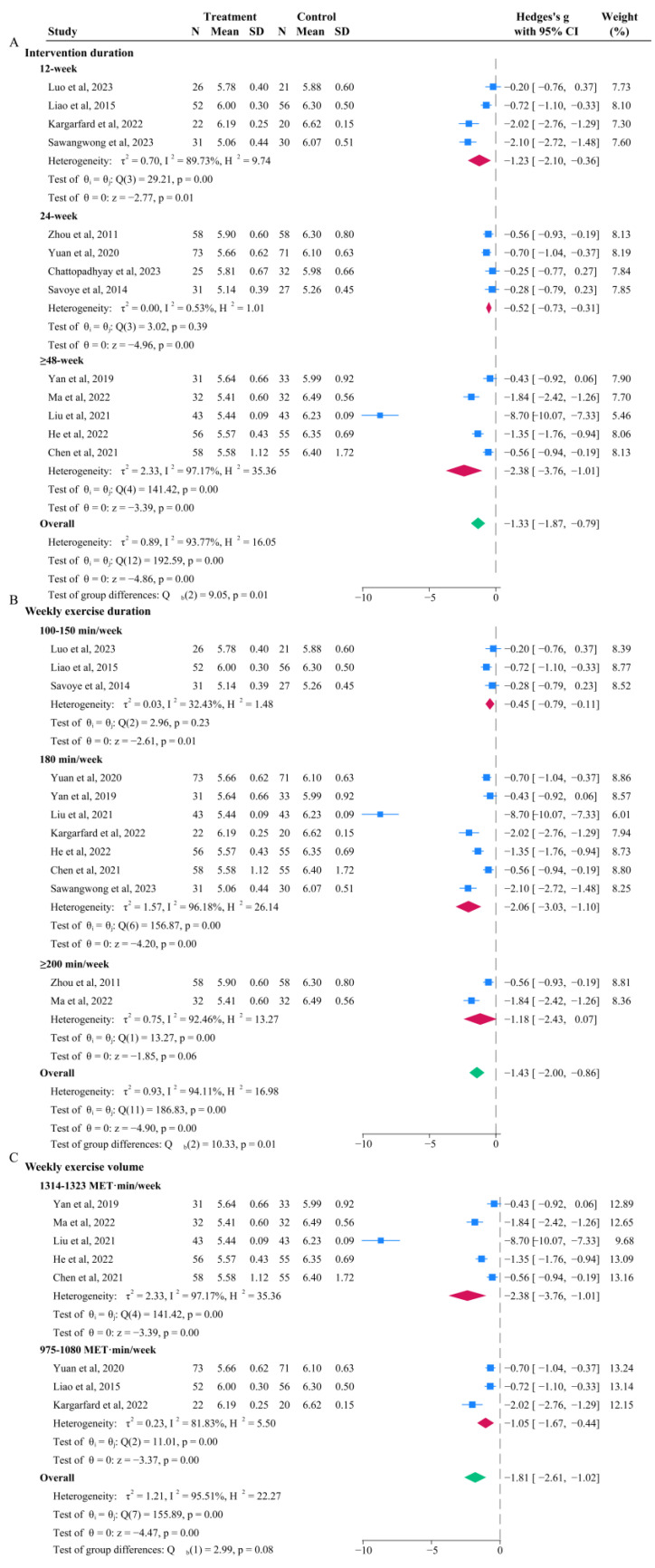
Forest plot for subgroup analysis of FBG [21,22,23,25,26,27,28,29,30,31,33,34,36]. (**A**) Based on intervention duration (week); (**B**) based on weekly exercise duration (min/week); (**C**) Based on weekly exercise volume (MET.min/week). Note: The ‘blue squares’ represent the effect size of each individual study, the ‘red diamonds’ represent the pooled value for the subgroup, and the ‘green diamond’ represents the overall pooled result for the entire group.

Among the 14 included studies, 13 compared the differences in FBG levels between the intervention group (*n* = 538) and control group (*n* = 533) (Figure 1A). The intervention group had significantly lower FBG levels compared to the control group (*p* < 0.001), with a pooled MD of −1.33 (95%CI: −1.87, −0.79). Subgroup analysis for FBG is shown in Figure 2. The pooled effect for ≥48-week group was significantly greater than that for 12-week and 24-week groups, and MD in the pooled effect for the 12-week group, 24-week group, and ≥48-week group was −1.23 (95% CI: −2.10, −0.36; *p* = 0.01), −0.52 (95% CI:−0.73, −0.31; *p* < 0.01), and −2.38 (95% CI:−3.76, −1.01; *p* < 0.01), respectively (Figure 2A). The pooled effect for the 180 min/week group and ≥200 min/week group was significantly greater than that for the 100–150 min/week group. The pooled MD for the 100–150 min/week group, 180 min/week group, and ≥200 min/week group was −0.45 (95% CI: −0.79, −0.11; *p* = 0.01), −2.06 (95% CI: −3.03, −1.10; *p* < 0.01), and −2.38 (95% CI: −3.76, −1.01; *p* = 0.06), respectively (Figure 2B). The pooled effect for the 1314–1323 MET·min/week group was greater than that for the 975–1080 MET·min/week group, but the difference was not statistically significant (*p* = 0.08). The pooled MD for the 975–1080 MET·min/week group and 1314–1323 MET·min/week group was −1.05 (95% CI: −1.67, −0.44; *p* < 0.01) and −2.38 (95% CI: −3.76, −1.01; *p* < 0.01), respectively (Figure 2C).

### 3.3. Effects of Aerobic Exercise on 2hPG

**Figure 3:**
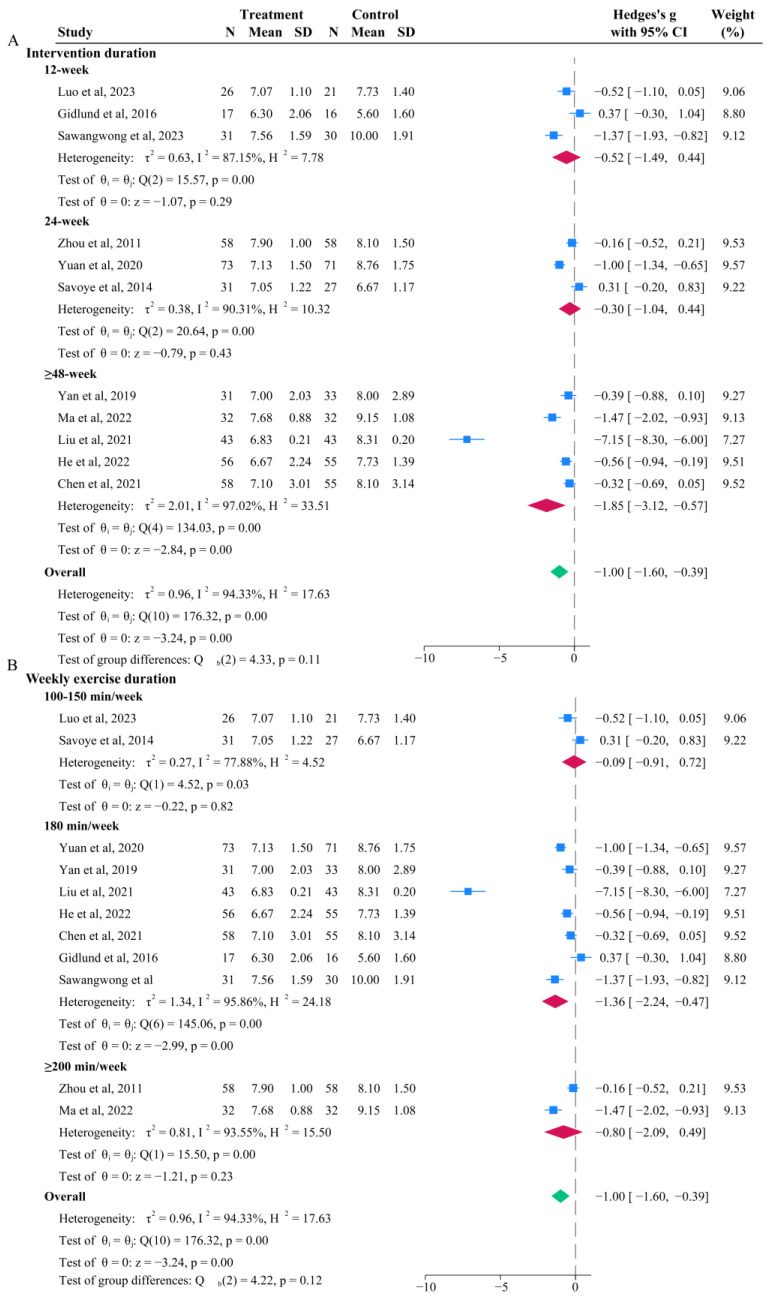
Forest plot for subgroup analysis of 2hPG [21,22,23,25,26,27,30,31,34,35,36]. (**A**) Based on intervention duration (week); (**B**) based on weekly exercise duration (min/week). Note: The ‘blue squares’ represent the effect size of each individual study, the ‘red diamonds’ represent the pooled value for the subgroup, and the ‘green diamond’ represents the overall pooled result for the entire group.

Among the 14 studies, 11 compared the difference in 2hPG levels between the intervention group (*n* = 456) and control group (*n* = 441) before and after intervention (Figure 1B). The intervention group had significantly lower 2hPG levels compared to the control group (*p* < 0.001), with an MD of −1.00 (95% CI: −1.60, −0.39). Subgroup analysis for 2hPG is shown in Figure 3. The pooled effect for the ≥48-week group was greater than that for 12-week and 24-week groups, but the difference was not statistically significant (Figure 3A). The pooled MD for the 12-week group, 24-week group, and ≥48-week group was −1.52 (95% CI: −1.49, −0.44; *p* = 0.29), −0.30 (95% CI: −1.04, −0.44; *p* = 0.43), and −1.85 (95%CI: −3.12, −0.57; *p* < 0.001), respectively. The pooled effect for the 180 min/week and ≥200 min/week groups was greater than that for the 100–150 min/week group, but the difference was not statistically significant (Figure 3B). The pooled MD for the 100–150 min/week group, 180 min/week group, and ≥200 min/week group was −0.09 (95% CI: −0.91, 0.72; *p* = 0.82), −1.36 (95% CI: −2.24, −0.47; *p* < 0.01), and −0.80 (95% CI: −2.09, 0.49; *p* = 0.23), respectively. The weekly exercise volume subgroup had less than two original studies.

### 3.4. Effects of Aerobic Exercise on HbA1c

**Figure 4:**
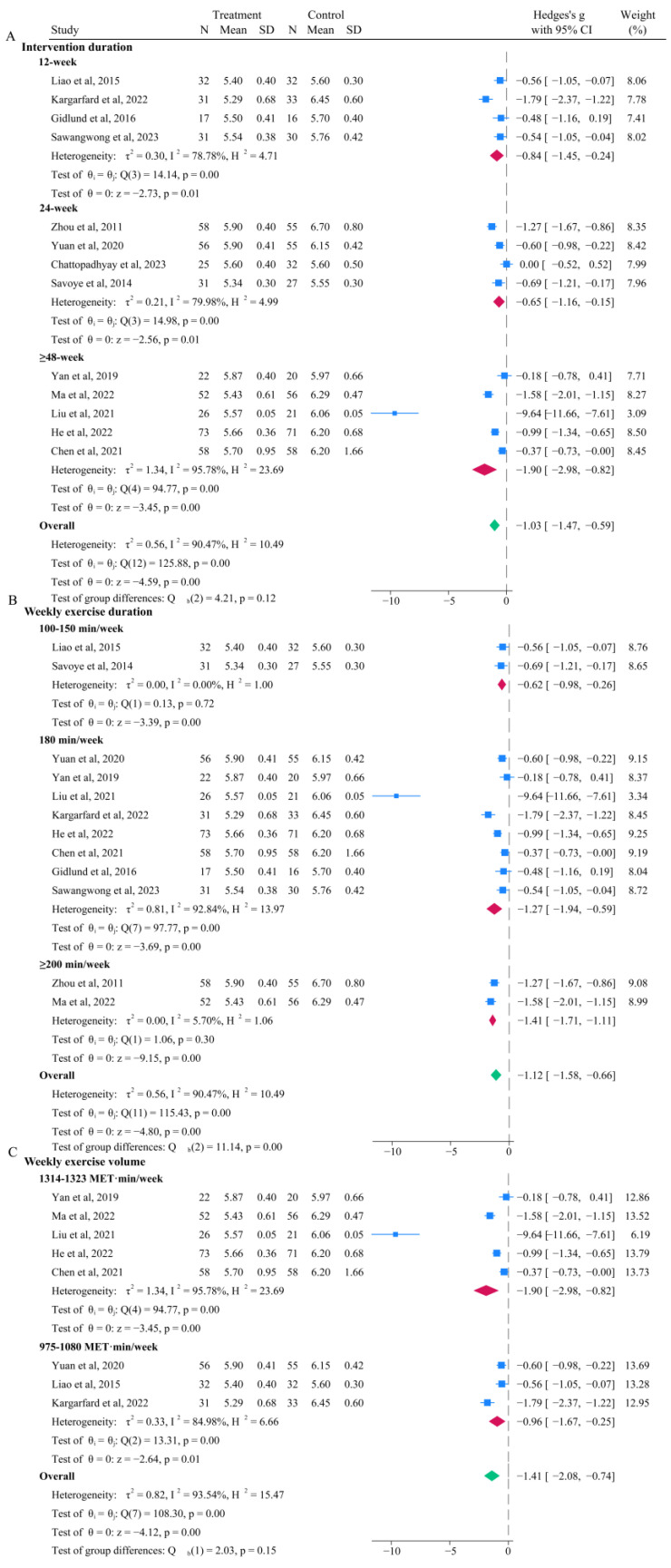
Forest plot for subgroup analysis of HbA1c [21,22,23,25,27,28,29,30,31,33,34,35,36]. (**A**) Based on intervention duration (week); (**B**) based on weekly exercise duration (min/week); (**C**) Based on weekly exercise volume (MET.min/week). Note: The ‘blue squares’ represent the effect size of each individual study, the ‘red diamonds’ represent the pooled value for the subgroup, and the ‘green diamond’ represents the overall pooled result for the entire group.

Among the 14 included studies, 13 compared the differences in HbA1c levels before and after intervention for patients with prediabetes, with the intervention group including 512 cases and the control group comprising 506 cases (Figure 1C). The intervention group had significantly lower HbA1c levels compared to the control group (*p* < 0.001), with an MD of −1.05 (95% CI: −1.49, −0.61) (Figure 4). Although the pooled effect for the ≥48-week group was greater than that for the 12-week and 24-week groups, the difference was not statistically significant. The pooled MD for the 12-week group, 24-week group, and ≥48-week group was −0.84 (95% CI: −1.45, −0.24; *p* = 0.01), −0.65 (95% CI: −1.16, −0.15; *p* = 0.01), and −1.90 (95% CI: −2.98, −0.82; *p* < 0.001), respectively (Figure 4A). The pooled effect for the ≥200 min/week group was significantly greater than that for the 100–150 min/week group and 180 min/week group, and the pooled MD for the 100–150 min/week group, 180 min/week group, and ≥200 min/week group was −0.62 (95%CI: −0.98, −0.26; *p* < 0.01), −1.27 (95%CI: −1.94, −0.59; *p* < 0.01), and −1.41 (95%CI: −1.71, −1.11; *p* < 0.01), respectively (Figure 4B). The pooled effect for the 1314–1323 MET·min/week group was significantly greater than that for the 975–1080 MET·min/week group, but the difference was not statistically significant (*p* = 0.15). The pooled MD for the 975–1080 MET·min/week group and 1314–1323 MET·min/week group was −0.96 (95% CI: −1.67, −0.25; *p* = 0.01) and −1.90 (95%CI: −2.98, −0.82; *p* < 0.01), respectively (Figure 4C).

### 3.5. Sensitivity Analysis

Sensitivity analyses were conducted for FBG, 2hPG, and HbA1C (Figure S2). Excluding individual studies did not change the significance of the pooled effect size, suggesting the stability and reliability of the results of this meta-analysis.

### 3.6. Quality Assessment

The results of the bias assessment are available in Figure S3. Among the included studies, 2 studies were assessed as low-risk, 11 studies showed some concerns, and 3 exhibited a high risk of bias (Figure S3A). Among the four evaluation domains, the randomization process had the highest proportion of some concerns, accounting for 50%. All studies were assessed as low risk regarding the measured outcomes, except for high risk regarding missing outcome data (Figure S3B).

### 3.7. Heterogeneity Analysis

The results of the heterogeneity test indicated that the I^2^ statistic for FPG, 2hPG, and HbA1c were greater than 50%, suggesting that heterogeneity may exist in this study. Publication bias tests were conducted for the 14 studies included in this meta-analysis. The funnel plots for FPG, 2hPG, and HbA1c can be found in Figure S4A–C. Although the funnel plots exhibited slight asymmetry, the results of Begg’s test were 0.246, 0.533, and 0.502 (all *p* > 0.05), respectively, indicating no evidence of publication bias.

## 4. Discussion

In this study, we conducted a systematic review and meta-analysis of randomized controlled trials implementing aerobic exercise interventions for individuals with prediabetes. The findings indicated that aerobic exercise can effectively reduce the risk of progression to diabetes among those with prediabetes. Specifically, it was shown that aerobic exercise positively affects fasting blood glucose, 2-h postprandial plasma glucose, and hemoglobin A1c, significantly contributing to blood glucose control. Subgroup analysis also demonstrated that a longer duration and volume of aerobic exercise within a specific timeframe can provide a greater improvement in blood glucose levels among those with prediabetes. Aerobic exercise can serve as a therapeutic approach for prediabetes and help reduce the risk of diabetes. Although this finding aligns with the well-established benefits of aerobic exercise on glucose control in diabetes, this study confirmed this effect in prediabetes, further highlighting its clinical significance.

Our study revealed that aerobic exercise protects individuals with prediabetes, effectively reducing FBG, 2hPG, and HbA1c levels. This effect on the detrimental effects of hyperglycemia aligns with previous findings [17,37]. Contrary to earlier reports suggesting the limited efficacy of exercise in lowering FBG [38,39], our results did not identify such limitations. Sensitivity analyses further underscored the reliability of our findings. Aerobic exercise enhances energy consumption, with elevated metabolic rates persisting for some time after exercise [40]. Additionally, aerobic exercise is generally linked to increased energy expenditure, maintaining healthy body weight, and improving lipid metabolism, although the exact mechanisms merit further explorations [41].

Patients with prediabetes typically exhibit impaired fasting glucose, impaired glucose tolerance, or both, often characterized by elevated or “inappropriately” increased production of endogenous glucose compared to normoglycemic individuals. This phenomenon originates from abnormalities in glucose regulation, including hepatic insulin resistance, reduced hepatic glucose clearance, and impaired glucose uptake responses. Delayed glucose uptake by skeletal muscle [42,43], varying degrees of insulin resistance, and β-cell dysfunction [44,45,46] are the common features of prediabetes. However, adherence to aerobic exercise in patients with prediabetes can effectively lower blood glucose levels, primarily by modifying glucose transporter 4 (GLUT4) translocation, skeletal muscle contraction, and fatty acid oxidation. Specifically, aerobic exercise can increase GLUT4 expression on muscle cells and enhance glucose uptake [47,48]. Additionally, skeletal muscle contraction promotes glucose consumption, aiding in blood glucose regulation [49,50]. Furthermore, aerobic exercise enhances fatty acid oxidation, reduces fat accumulation, and improves insulin resistance [50,51,52]. In summary, aerobic exercise protects individuals with prediabetes by improving insulin sensitivity and metabolic functions, making it a promising intervention for managing prediabetes.

In this study, subgroup analysis indicated that longer durations of intervention, greater weekly exercise duration, and higher exercise volumes are associated with greater effects. Prolonged aerobic exercise may induce multifaceted physiological alterations, improve insulin sensitivity, and enhance energy metabolism, leading to more effective reductions in blood glucose levels among individuals with prediabetes. Consequently, longer exercise durations may demonstrate more significant advantages in regulating blood glucose levels. However, after subgrouping based on exercise durations, we found that the difference in the improvement of blood glucose levels between exercise over 48 weeks and other exercise durations was not statistically significant. In the early stages of exercise, aerobic exercise induces positive physiological adaptations; for instance, it improves cardiovascular function and increases insulin sensitivity, which in turn results in a rapid decline in blood glucose levels [50,53]. The adaptive effect gradually saturates as the exercise duration increases; thus, the physiological systems no longer exhibit significant improvement, leading to decreased efficacy. Therefore, once the effect of exercise on blood glucose is stabilized, physiological systems can be further stimulated by intensifying or extending exercise [24]. Furthermore, previous studies have shown that the organism maintains the stability of the internal environment through regulatory mechanisms, and exercise beyond a certain duration or intensity may induce adaptive homeostasis, thereby attenuating the regulatory effect of exercise on blood glucose [47]. Therefore, when formulating exercise plans, it is necessary to adjust the intensity and duration of exercise based on patients’ blood glucose level to achieve the optimal effect on prediabetes.

While this meta-analysis provided evidence for the effectiveness of aerobic exercise in the management of blood glucose levels among individuals with prediabetes, translating this knowledge into practical recommendations is essential for broader application. Therefore, the recommendation should not simply be to “increase aerobic exercise”, but it should encourage selecting a form of exercise that is both enjoyable and accessible to individuals [54]. Several options are available for cyclic endurance activities, such as brisk walking, jogging, cycling, or swimming. The studies included in this analysis primarily focused on moderate-intensity exercise, which can be defined as an activity that induces some extent of breathlessness but still allows the individual to maintain a conversation [13]. Aquatic exercises, such as water walking and water aerobics, may be a suitable choice for individuals with joint pain or other physical limitations, as the buoyancy of water reduces joint load [55]. Exercise programs should be individualized, considering the specific mode, duration, intensity, and frequency. These parameters should gradually increase over time to ensure both safety and sustained adherence. Finally, individuals should consult with their healthcare provider before initiating any new exercise program and select an enjoyable and practical mode, which can promote long-term adherence.

Nevertheless, our findings exhibited substantial heterogeneity, introducing uncertainty to the pooled results. Heterogeneity persisted despite conducting subgroup analyses. Notably, 9 out of the 14 studies included in this meta-analysis were from China, thereby mitigating the impact of ethnic diversity on heterogeneity [56]. Although most of the fourteen studies controlled exercise intensity within 60–70% of HRmax and exercise frequency at three times per week, there was considerable diversity in the types of exercises employed (e.g., jogging, yoga, dancing, and brisk walking), which led to significant heterogeneity. Additionally, the age and gender distribution of the study populations might affect the results. For instance, Savoye et al. included participants with a relatively lower average age (around 12 years), but other studies included individuals aged 60 years and above [34]. In the study conducted by Liu et al., there was a marked gender imbalance, with more than 90% of participants being female. In addition, in this study, the standard deviation of each blood glucose level indicator after intervention was significantly lower than that reported by other studies [27]. Similarly, high heterogeneity was observed in other similar studies [39,57,58], possibly due to substantial differences in demographic information, exercise modalities, intervention durations, and participant characteristics. Nevertheless, all studies consistently demonstrated that aerobic exercise can significantly decrease blood glucose levels, suggesting that aerobic exercise can effectively prevent the progression from prediabetes to diabetes.

This meta-analysis of RCTs has inherent risks of bias, with the greatest concerns observed in the randomization process. The primary issue originated from the lack of detailed elaboration on the random allocation process and the concealment of group assignments. Specifically, four studies claimed to be “randomized”, yet they did not further elaborate on the randomization process [21,23,28,29]. Moreover, many studies lacked explicit descriptions about concealment [23,26,27,28,29,30,31]; thus, they were categorized as “no information”. Regarding deviations from the intended interventions, bias was introduced as participants and implementers of interventions were aware of group assignments because there were distinct differences between the intervention group and the control group, which regularly engaged in aerobic exercises of a specified intensity each week. Additionally, studies with follow-up losses necessitated intention-to-treat analyses, and failure to conduct such analyses was deemed unreasonable. Although the missing outcome data generally indicated a low risk of bias, almost all studies with outcome data included participants accounting for 95% of the population at the beginning of follow-up. Three studies were deemed high risk due to potential associations between participant attrition and their health conditions [31,34,59]. The measurement of the outcome domain demonstrated low risks of bias, as the measurement methods were well-established, and the outcome assessment methods were appropriate and comparable. Finally, nine studies were registered for clinical trials [23,24,26,27,29,30,31,32,36]. Overall, the quality of the included studies was relatively high.

Recently, exercise therapy has been widely recognized as a cost-effective, user-friendly, and safe intervention for the prevention or treatment of diabetes, distinguishing itself from other approaches [60]. However, existing evidence suggests that aerobic exercise, or exercise in general, may have limited efficacy in controlling blood glucose. For instance, Jiang et al. [38] indicated that exercise intervention did not significantly affect FBG. Similarly, Zheng et al. [39] suggested that exercise alone has limited efficacy in reducing FBG in individuals with prediabetes. In contrast, exercise combined with dietary intervention can significantly lower FPG levels. A consensus statement from the American College of Sports Medicine proposed that interventions combining aerobic exercise and resistance training may outcompete any single modality alone [53]. These studies collectively suggest that singular aerobic exercise (or exercise) has limited efficacy in blood glucose control, and a combination of various approaches, such as diet, lifestyle modification, and medications, are needed to achieve better outcomes.

Our study had several limitations. First, the study did not differentiate between patients with IGT and IFG. IGT and IFG have certain differences in their pathogenesis and progression to diabetes, which makes the study population heterogeneous. Second, similar to other studies [39,57,58], heterogeneity was observed across the included studies in this meta-analysis, which may increase the uncertainty of the pooled results. Third, due to the relatively strict inclusion and exclusion criteria, the number of RCTs included in this meta-analysis was limited. Most studies were from China, which might lead to potential regional and ethnic biases. The included studies lacked data on dietary intake, supplement use, and specific indicators (e.g., waist-to-hip ratio and high-sensitivity C-reactive protein), all of which can affect blood glucose levels and the development of diabetes. Future studies should address these factors to more comprehensively understand the effects of exercise on blood glucose levels.

## 5. Conclusions

This study highlighted that aerobic exercise can effectively lower blood glucose levels and reduce the risk of diabetes among individuals with prediabetes. The volume and duration of aerobic exercise are important factors affecting blood glucose levels, exhibiting a positive correlation. Notably, the intensity and duration of aerobic exercise should be adjusted based on patients’ blood glucose levels tooptimize the management of prediabetes. Further high-quality, long-term RCT studies are needed to obtain more precise and detailed findings.

## Figures and Tables

**Table 1:** Characteristics of included studies that incorporated prediabetes interventions.

First Author, Year	Sample Size	Age (Years, Mean ± SD)	Gender (F/M)	Exercise Type	Exercise Frequency	Exercise Duration (min/Day)	Weekly Exercise Duration (min/Week)	Weekly Exercise Volume (MET.min/Week)	Intervention Duration	Exercise Intensity	Classification of Exercise Type
Zhou et al., 2011 [21]	58	65.6 ± 11.9	36/22	Aerobic exercise methods such as walking, jogging, boxing, dancing, etc.	≥5 times/week	≥40 min/day	≥200	/	24 weeks	Low strength	Acyclic
Yuan et al., 2020 [22]	83	60.93 ± 5.71	59/24	Jogging	3 times/week	60 min/day	180	1080	24 weeks	HRmax 60–70%	Cyclic
Yan et al., 2019 [23]	35	64.23 ± 5.75	25/10	Aerobic exercises (aerobic dancing)	3 times/week	60 min/day	180	1314	48 weeks	HRmax 60–70%	Acyclic
Slentz et al., 2016 [24]	61	\	\	Treadmills, cycle, and rowing	6 h/week	/	360	42	24 weeks	50%VO2; 75%VO2	Cyclic
Ma et al., 2022 [25]	44	\	\	Moderate-intensity aerobic exercise, mainly rhythmic exercises and square dances	Once/2 day	60 min/day	210	1323	48 weeks	HRmax 40–60%	Acyclic
Luo et al., 2023 [26]	27	\	\	Walking or running, combined with aerobic gymnastics and swimming	3 times/week	50 min/day	150	/	12 weeks	40–49% VO2 (1–4); 50–59% VO2 (5–12)	Cyclic
Liu et al., 2021 [27]	43	60.35 ± 4.29	40/3	Aerobic dancing	3 times/week	60 min/day	180	1314	48 weeks	HRmax 60–70%	Acyclic
Liao et al., 2015 [28]	60	42.4 ± 5.8	27/33	Moderate aerobic exercise (jogging or brisk walking)	≥5 times/week	≥30 min/day	≥150	≥975	12 weeks	moderate intensity	Cyclic
Kargarfard et al., 2022 [29]	25	\	\	Walking and jogging on treadmill	3 times/week	60 min/day	180	1080	12 weeks	HRmax 50–60%	Cyclic
He et al., 2022 [30]	83	60.93 ± 5.71	59/24	Aerobic exercises (dancing with music)	3 times/week	60 min/day	180	1314	96 weeks	HRmax 60–70%	Acyclic
Chen et al., 2021 [31]	83	60.93 ± 5.71	59/24	Aerobic dancing	3 times/week	60 min/day	180	1314	96 weeks	HRmax 60–70%	Acyclic
Badaam et al., 2021 [32]	80	30.7 ± 3.3	\	Moderate intensity (brisk walking)	5 times/week	30 min/day	150	975	12 weeks	Moderate intensity	Cyclic
Chattopadhyay et al., 2023 [33]	25	41.3 ± 7.4	\	Yoga	2 times/week	45 min/day (1–4 week) 75 min (5 week+)	/	/	24 weeks	Low intensity	other
Savoye et al., 2014 [34]	31	12.7 ± 1.9	21/10	A warm-up, high-intensity, and cool-down period. High-intensity exercises consisted of typical children’s games	2 times/week	50 min/day	100	/	24 weeks	High intensity	other
Gidlund et al., 2016 [35]	17	56 ± 5.6	0/17	Nordic Walking	3 times/week	60 min/day	180	/	12 weeks	1–4 week at 55%, 5–8 week at 65%, and 9–12 week at 75%	Cyclic
Sawangwong et al., 2023 [36]	31	49.31 ± 13.01	8/23	Traditional Thai Exercise (Ruesi Dadton)	3 times/week	60 min/day	180	/	12 weeks	Low intensity	other

Notes: SD, standard deviation; F/M, female/male.

## Data Availability

All data generated or analyzed during this study are included in this published article and its Supplementary Materials. The original data can be obtained by the reasonable application of the corresponding author.

## Supplementary Materials

The following supporting information can be downloaded at: https://www.mdpi.com/article/10.3390/life15010032/s1, Figure S1: The PRISMA (2020) flowchart of study selection; Table S1: PRISMA 2020 Checklist; Figure S2. Sensitivity analysis; Figure S3. The results of biased risk assessment; Figure S4. Publication bias.

## Abbreviations

2hPG, 2-h postprandial plasma glucose; 95% CI, 95% Confidence interval; FBG, fasting blood glucose; HbA1c, hemoglobin A1c; MD, Mean Difference; MET, Metablic equivalent; RCT, randomized controlled trial; PRISMA, Preferred Reporting Items for Systematic Reviews and Meta-Analyses; PROSPERO, Prospective Register of Systematic Reviews; SD, standard deviation.
