# Supplementary material for: Effect of Aerobic Exercise on Blood Glucose Among Those with Prediabetes: A Systematic Review and Meta-Analysis
Source: Life (Basel). 2024 Dec 30;15(1):32. doi: 10.3390/life15010032 (PMC11766620; doi:10.3390/life15010032)

Supplement Material

Effect of Aerobic Exercise on Blood Glucose Among Those with Prediabetes: A Systematic Review and Me-ta-Analysis

Content

Supplementary material S1: PRISMA 2020 Checklist. 2
Supplementary Material S2: Search terms and search strategy. 6
Supplementary Material S3: The result of data pooling. 8

Supplementary material S1: PRISMA 2020 Checklist.

Table S1: PRISMA 2020 Checklist.

Section and Topic	Item #	Checklist item	Location where item is reported
**TITLE**			
Title	1	Identify the report as a systematic review.	Page 1
**ABSTRACT**			
Abstract	2	See the PRISMA 2020 for Abstracts checklist.	Page 1
**INTRODUCTION**			
Rationale	3	Describe the rationale for the review in the context of existing knowledge.	Page 2
Objectives	4	Provide an explicit statement of the objective(s) or question(s) the review addresses.	Page 2
**METHODS**			
Eligibility criteria	5	Specify the inclusion and exclusion criteria for the review and how studies were grouped for the syntheses.	Page 3
Information sources	6	Specify all databases, registers, websites, organisations, reference lists and other sources searched or consulted to identify studies. Specify the date when each source was last searched or consulted.	Page 3
Search strategy	7	Present the full search strategies for all databases, registers and websites, including any filters and limits used.	Supplementary Material 2
Selection process	8	Specify the methods used to decide whether a study met the inclusion criteria of the review, including how many reviewers screened each record and each report retrieved, whether they worked independently, and if applicable, details of automation tools used in the process.	Page 3
Data collection process	9	Specify the methods used to collect data from reports, including how many reviewers collected data from each report, whether they worked independently, any processes for obtaining or confirming data from study investigators, and if applicable, details of automation tools used in the process.	Page3
Data items	10a	List and define all outcomes for which data were sought. Specify whether all results that were compatible with each outcome domain in each study were sought (e.g. for all measures, time points, analyses), and if not, the methods used to decide which results to collect.	Page 3, Data extraction
	10b	List and define all other variables for which data were sought (e.g. participant and intervention characteristics, funding sources). Describe any assumptions made about any missing or unclear information.	Page 3, Data extraction
Study risk of bias	11	Specify the methods used to assess risk of bias in the included studies, including details of the tool(s) used, how many reviewers assessed	Page 3-4, Quality assessment

Section and Topic	Item #	Checklist item	Location where item is reported
assessment		each study and whether they worked independently, and if applicable, details of automation tools used in the process.	
Effect measures	12	Specify for each outcome the effect measure(s) (e.g. risk ratio, mean difference) used in the synthesis or presentation of results.	Page 4, Statistical analysis
Synthesis methods	13a	Describe the processes used to decide which studies were eligible for each synthesis (e.g. tabulating the study intervention characteristics and comparing against the planned groups for each synthesis (item #5)).	Page 3, Data extraction
	13b	Describe any methods required to prepare the data for presentation or synthesis, such as handling of missing summary statistics, or data conversions.	Page 4, Statistical analysis
	13c	Describe any methods used to tabulate or visually display results of individual studies and syntheses.	Page 4, Statistical analysis
	13d	Describe any methods used to synthesize results and provide a rationale for the choice(s). If meta-analysis was performed, describe the model(s), method(s) to identify the presence and extent of statistical heterogeneity, and software package(s) used.	Page 4, Statistical analysis
	13e	Describe any methods used to explore possible causes of heterogeneity among study results (e.g. subgroup analysis, meta-regression).	Page 4, Statistical analysis; 'Additional efforts were explored to identify sources of heterogeneity in three separate subgroup meta-analyses'.
	13f	Describe any sensitivity analyses conducted to assess robustness of the synthesized results.	Page 4, Statistical analysis; 'Heterogeneity was assessed using the I^2 statistic and Spearman's correlation coefficient'
Reporting bias assessment	14	Describe any methods used to assess risk of bias due to missing results in a synthesis (arising from reporting biases).	Page 4, 'Publication bias was visually assessed using funnel plots'.
Certainty assessment	15	Describe any methods used to assess certainty (or confidence) in the body of evidence for an outcome.	Page 4, All results were harmonized into the format "mean ± standard deviation (M ± SD)" for data synthesis.

Section and Topic	Item #	Checklist item	Location where item is reported
**RESULTS**			
Study selection	16a	Describe the results of the search and selection process, from the number of records identified in the search to the number of studies included in the review, ideally using a flow diagram.	Page 4 Study selection and Figure S1
	16b	Cite studies that might appear to meet the inclusion criteria, but which were excluded, and explain why they were excluded.	NR
Study characteristics	17	Cite each included study and present its characteristics.	Page 4, Characteristics of Eligible Studies, Table 1
Risk of bias in studies	18	Present assessments of risk of bias for each included study.	Page 14, Quality Assessment
Results of individual studies	19	For all outcomes, present, for each study: (a) summary statistics for each group (where appropriate) and (b) an effect estimate and its precision (e.g. confidence/credible interval), ideally using structured tables or plots.	Page 7-14, Figure 1-4
Results of syntheses	20a	For each synthesis, briefly summarise the characteristics and risk of bias among contributing studies.	Page 4, Characteristics of Eligible Studies, Table 1; Page 6, Quality assessment.
	20b	Present results of all statistical syntheses conducted. If meta-analysis was done, present for each the summary estimate and its precision (e.g. confidence/credible interval) and measures of statistical heterogeneity. If comparing groups, describe the direction of the effect.	Page 7-14, Figure 1-4
	20c	Present results of all investigations of possible causes of heterogeneity among study results.	Page 14, Heterogeneity analysis
	20d	Present results of all sensitivity analyses conducted to assess the robustness of the synthesized results.	Page 14, Sensitivity Analysis
Reporting biases	21	Present assessments of risk of bias due to missing results (arising from reporting biases) for each synthesis assessed.	Page 14, Quality Assessment
Certainty of evidence	22	Present assessments of certainty (or confidence) in the body of evidence for each outcome assessed.	Page 14, Sensitivity Analysis
**DISCUSSION**			
Discussion	23a	Provide a general interpretation of the results in the context of other evidence.	Page 14
	23b	Discuss any limitations of the evidence included in the review.	Page 14-17
	23c	Discuss any limitations of the review processes used.	Page 17 the last paragraph of the

Section and Topic	Item #	Checklist item	Location where item is reported
			Discussion
	23d	Discuss implications of the results for practice, policy, and future research.	Page 17, Conclusions
**OTHER INFORMATION**			
Registration and protocol	24a	Provide registration information for the review, including register name and registration number, or state that the review was not registered.	Page 3, 'This study is registered with PROSPERO, CRD42024490348'.
	24b	Indicate where the review protocol can be accessed, or state that a protocol was not prepared.	N/A
	24c	Describe and explain any amendments to information provided at registration or in the protocol.	N/A
Support	25	Describe sources of financial or non-financial support for the review, and the role of the funders or sponsors in the review.	Page 17
Competing interests	26	Declare any competing interests of review authors.	Page 17, Declaration of competing interest
Availability of data, code and other materials	27	Report which of the following are publicly available and where they can be found: template data collection forms; data extracted from included studies; data used for all analyses; analytic code; any other materials used in the review.	Page 17, Data availability

From: Page MJ, McKenzie JE, Bossuyt PM, Boutron I, Hoffmann TC, Mulrow CD, et al. The PRISMA 2020 statement: an updated guideline for reporting systematic reviews. BMJ 2021;372:n71. doi: 10.1136/bmj.n71

For more information, visit: http://www.prisma-statement.org/

Supplementary Material S2: Search terms and search strategy.

(1) PubMed

#1 prediabetic state[MeSH]

#2 (prediabet*[Title/Abstract]) OR (impaired fasting gl*[Title/Abstract]) OR (impaired glucose tolerance[Title/Abstract]) OR (glucose intolerance[Title/Abstract]) OR (impaired glucose regulation[Title/Abstract]) OR (glucose metabolism disorders[Title/Abstract]) OR (IGT[Title/Abstract]) OR (IFG[Title/Abstract]) OR (IGR[Title/Abstract])

#3 #1 OR #2

#4 exercise[MeSH]

#5 (exercise[Title/Abstract]) OR (aerobic*[Title/Abstract]) OR (cardio[Title/Abstract])

#6 #4 OR #5

#7 ((randomized controlled trial[Publication Type]) OR (controlled clinical trial[Publication Type]))

#8 (randomized clinical trial*[Title/Abstract]) OR (randomized controlled trial*[Title/Abstract]) OR (randomized experiment[Title/Abstract]) OR (RCT[Title/Abstract])

#9 #7AND #8

#10 #3 and #6 and #9

(2) Embase

#1 'impaired glucose tolerance'/exp

#2 'prediabet*':ab,kw,ti OR 'impaired fasting gl*':ab,kw,ti OR 'impaired glucose tolerance':ab,kw,ti OR 'glucose intolerance':ab,kw,ti OR 'impaired glucose regulation':ab,kw,ti OR 'glucose metabolism disorders':ab,kw,ti OR 'IGT':ab,kw,ti OR 'IFG':ab,kw,ti OR 'IGR':ab,kw,ti

#3 #1 OR #2

#4 'aerobic exercise'/exp

#5 'exercise':ab,kw,ti OR 'aerobic*':ab,kw,ti OR 'cardio':ab,kw,ti

#6 #4 OR #5

#7 'randomized controlled trial'/exp OR 'controlled clinical trial'/exp

#8 'randomized clinical trial*':ab,kw,ti OR 'randomized controlled trial*':ab,kw,ti OR 'randomized experiment':ab,kw,ti OR RCT:ab,kw,ti

#9 #7 OR #8

#8 #3 AND #6 AND #9

(3) The Cochrane Library

#1 MeSH descriptor: [Prediabetic State] explode all trees

#2 (IGT):ti,ab,kw OR (IFG):ti,ab,kw OR (IGR):ti,ab,kw OR (glucose metabolism disorders):ti,ab,kw OR (impaired fasting gl*):ti,ab,kw OR (impaired glucose tolerance):ti,ab,kw OR (prediabet*):ti,ab,kw OR (glucose Intolerance):ti,ab,kw OR (impaired glucose regulation):ti,ab,kw):ti,ab,kw

#3 #1 or #2

#4 MeSH descriptor: [Exercise] explode all trees

#5 (Exercise):ti,ab,kw OR (aerobic*):ti,ab,kw OR (cardio):ti,ab,kw

#6 #4 or #5

#7 MeSH descriptor: [Randomized Controlled Trial] explode all trees

#8 (randomized clinical trial*):ti,ab,kw OR (randomized controlled trial*):ti,ab,kw OR (randomized experiment):ti,ab,kw OR (RCT):ti,ab,kw

#9 #7 OR #8

#10 #3 AND #6 AND #9

(4) Web of science Core Collection

(TS=(prediabet* or impaired fasting gl * or impaired glucose tolerance or glucose intolerance or impaired glucose regulation or glucose metabolism disorders or IGT or IFG or IGR)) and (TS=(exercise or aerobic* or cardio)) and(TS=(randomized clinical trial* or randomized controlled trial* or randomized experiment or RCT))

(5) CINAHL Plus with Full Text

#1 (MH "Prediabetic State")

#2 SU prediabet* OR SU impaired fasting gl* OR SU impaired glucose tolerance OR SU glucose intolerance OR SU impaired glucose regulation OR SU glucose metabolism disorders OR SU IGT OR SU IFG OR SU IGR

#3 #1 OR #2

#4 (MH "Aerobic Exercises")

#5 SU exercise OR SU aerobic* OR SU cardio

#6 #4 OR #5

#7 (MH "Randomized Controlled Trials+")

#8 SU randomized clinical trial* OR SU randomized controlled trial* OR SU randomized experiment OR SU RCT

#9 #7 OR #8

#10 #3 AND #6 AND #9

Supplementary Material S3: The result of data pooling.

Figure S1: The PRISMA (2020) flowchart of study selection.

Displayed is the PRISMA (preferred reporting items for systematic reviews and meta-analyses) flow of search methodology and literature selection process.

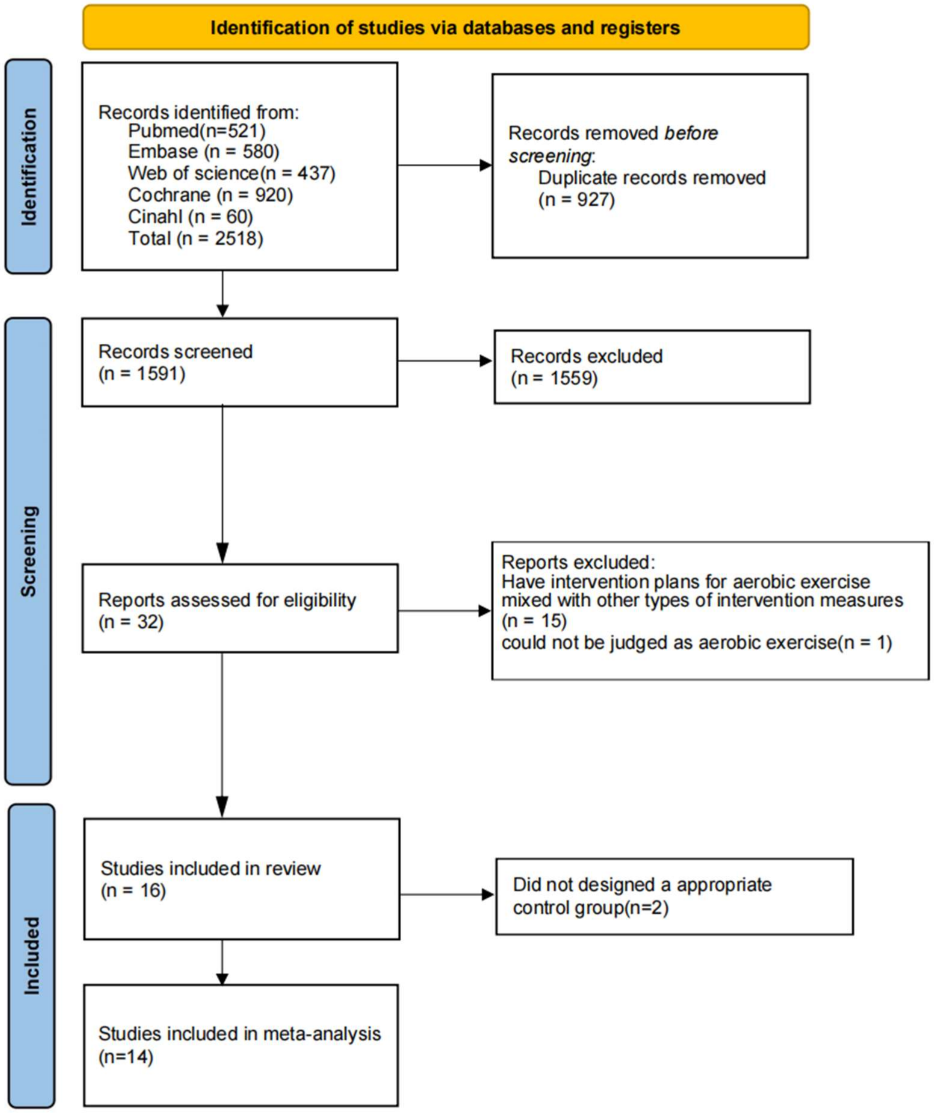

Figure S2. Sensitivity analysis.

A. The sensitivity analysis of FBG;

B. The sensitivity analysis of 2hPG;

C. The sensitivity analysis of Hba1c.

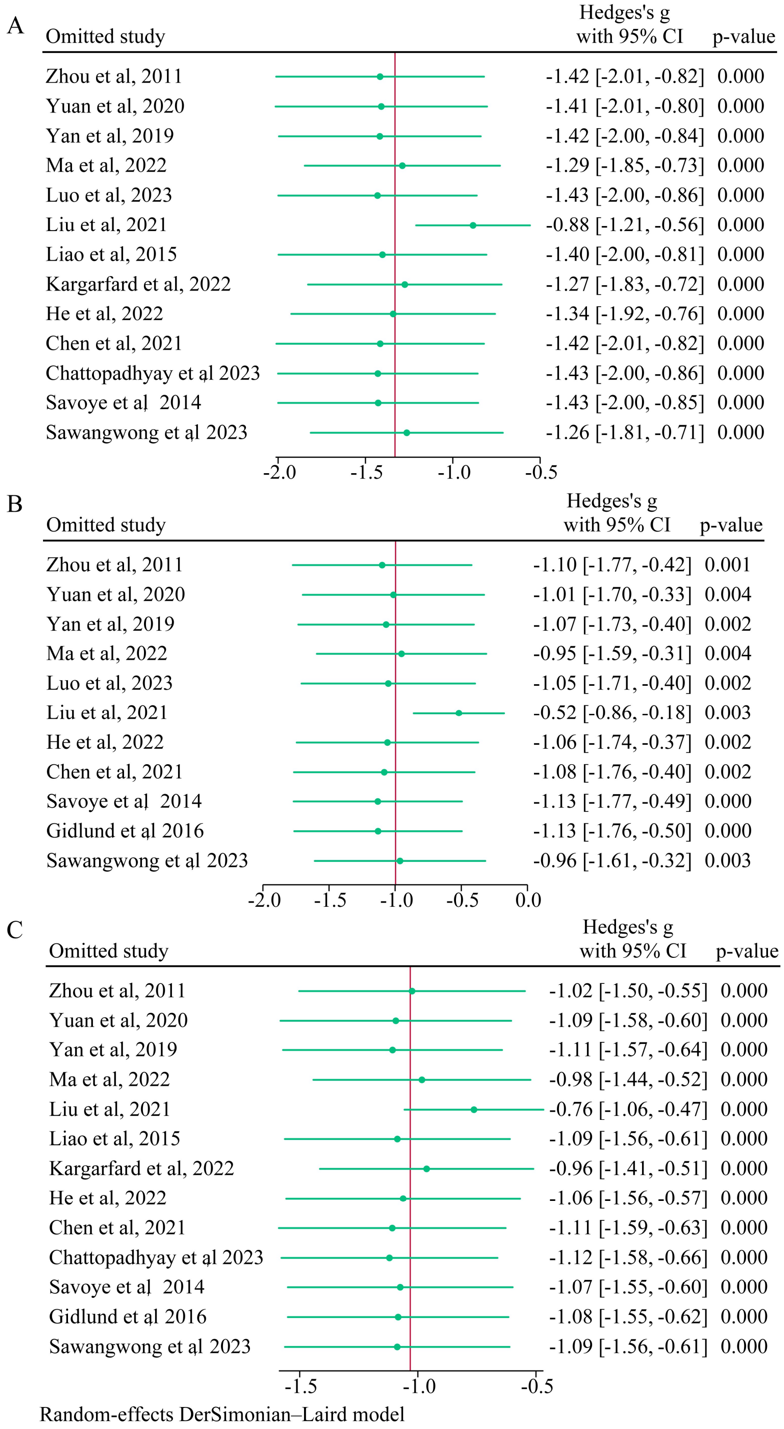

Figure S3. The results of biased risk assessment.

A. The risk of bias graph: review authors' judgements about each risk of bias item presented as percentages across all included 16 studies;

B. The risk of bias summary: review authors' judgements about each risk of bias item for each included study.

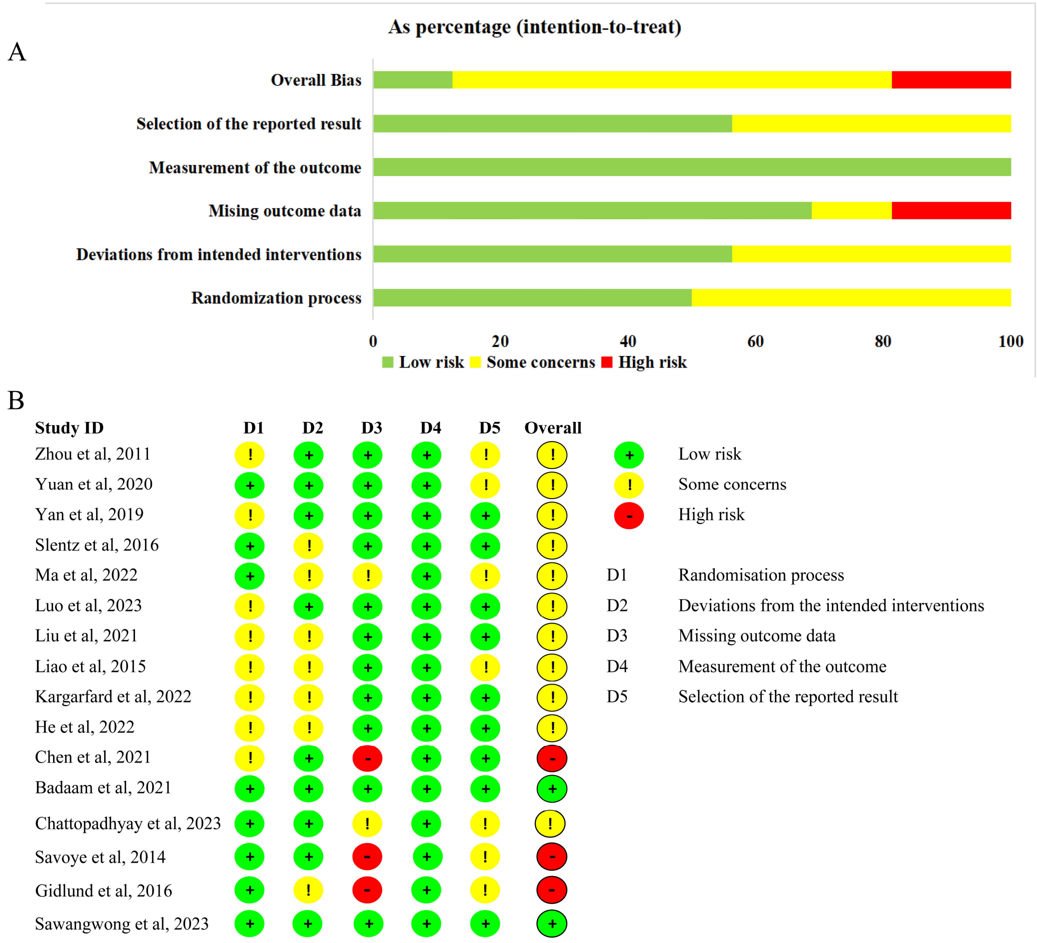

Risk of bias (ROB) report of included studies using Cochrane's ROB 2.0, and its adapted version for cluster RCTs.

Figure S**4.** Publication bias.

A. The Begg's funnel diagram of FBG;

B. The Begg's funnel diagram of 2hPG;

C. The Begg's funnel diagram of HbA1C.

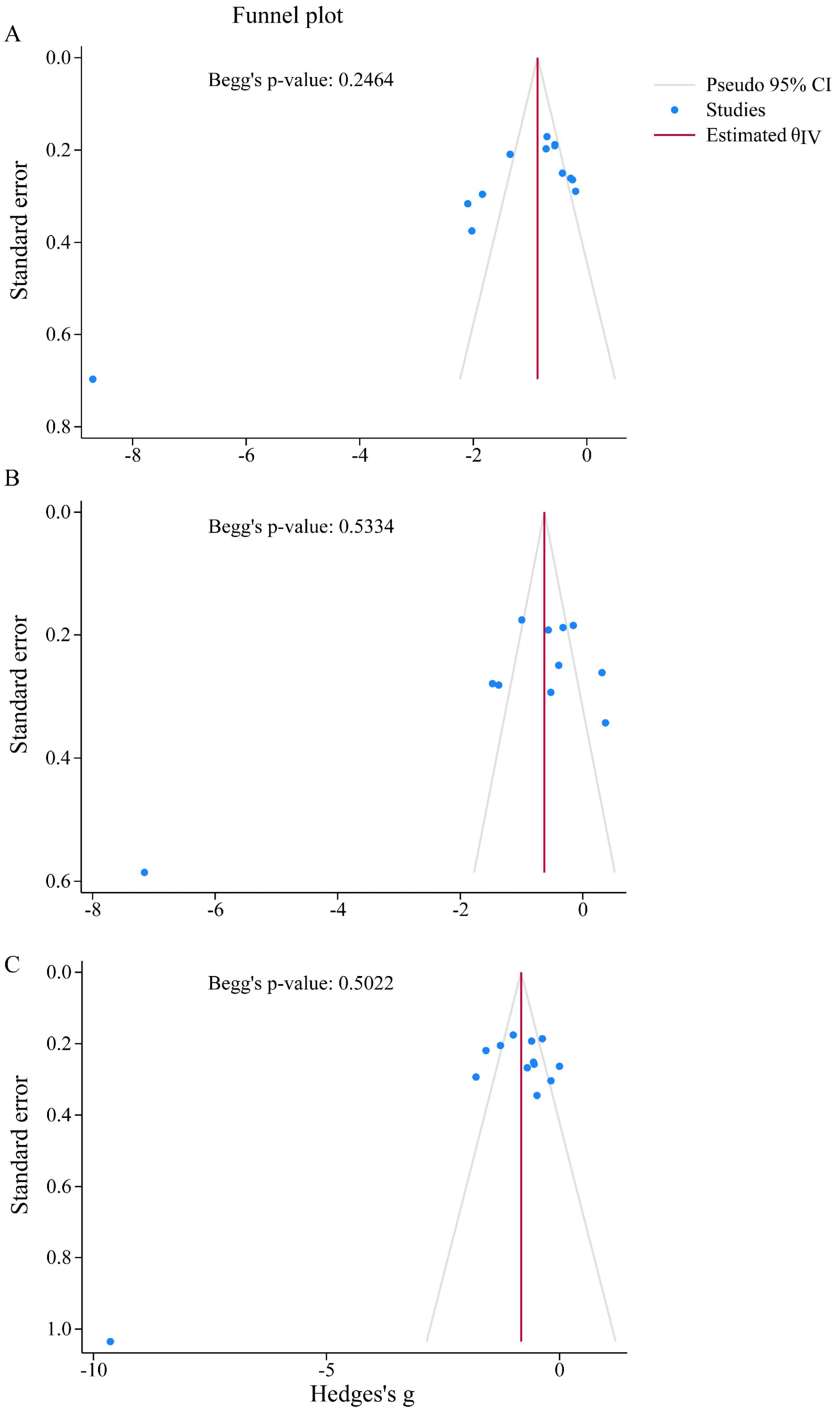

Supplement: Supplementary file 1 [file life-15-00032-s001.zip › life-3309542-supplementary.pdf]
